# Effectiveness of New Isomalt-Containing Toothpaste Formulations in Preventing Dental Caries: A Microbial Study

**DOI:** 10.3390/dj12090290

**Published:** 2024-09-12

**Authors:** Bennett Tochukwu Amaechi, Parveez Ahamed Abdul Azees, Sahar Mohseni, Maria Camila Restrepo-Ceron, Yuko Kataoka, Temitope Olabisi Omosebi, Kannan Kanthaiah

**Affiliations:** 1 Department of Comprehensive Dentistry, School of Dentistry, University of Texas Health San Antonio, San Antonio, TX 78712, USA; abdulazees@uthscsa.edu (P.A.A.A.); mohseni@uthscsa.edu (S.M.); mc.restrepo4@gmail.com (M.C.R.-C.); daisukiarao@gmail.com (Y.K.); kanthaiah@uthscsa.edu (K.K.); 2 Department of Odontologia, School of Odontologa, CES University, Medellín 050001, Colombia; 3 Department of Restorative Dentistry, Lagos State University Teaching Hospital, Ikeja 100271, Nigeria; topeisokay@yahoo.com

**Keywords:** isomalt, toothpaste, caries prevention, fluoride, demineralization

## Abstract

This study investigated the efficacy of Isomalt-containing toothpaste in preventing development of dental caries. Methods: Human dental enamel slabs were allocated to six groups (30/group) at random: De-ionized distilled water (DDW), and toothpaste containing 10% Isomalt, 1100 ppm fluoride, 0.05% cetylpyridinium chloride [CPC] (ICT); 10% Isomalt, 1100 ppm fluoride (IT); 10% Isomalt, 1100 ppm fluoride, 1.5% Sodium lauryl sulfate [SLS] (IST); 1100 ppm fluoride only (FT); 1100 ppm fluoride with SLS (FST). The enamel slabs were exposed to caries development via plaque growth in a Microbial Caries Model for 7 days. Toothpastes were applied as slurries (one toothpaste–three DDW) for 2 min twice daily. Demineralization was measured as the change in surface microhardness (ΔSMH) and amount of mineral lost (∆Z), and these metrics were assessed using Transverse Microradiography. Intra-group (SMH) and intergroup (%∆SMH and ∆Z) comparisons were paired *t*-test and Tukey’s test (α = 0.05), respectively. Results: With SMH, demineralization was found to be significant (*p* < 0.001) in all groups compared to sound enamel baseline, except ICT group. With %ΔSMH, all other groups had significantly (*p* < 0.001) less demineralization compared to DDW. Significantly (*p* < 0.001) greater demineralization was observed in IT, FT and FST compared to ICT, and no significant difference was observed between IST and ICT or FT. With ∆Z, relative to the DDW group, the inhibition of demineralization was significant (*p* < 0.0001) in all groups at varying percentages. Conclusions: Toothpaste containing 10% Isomalt, 1100 ppm fluoride, and 0.05% CPC demonstrated greater efficacy in inhibiting caries development amid dental plaque compared to toothpaste containing only 1100 ppm fluoride.

## 1. Introduction

Despite the development of preventive and operative approaches for caries management, the prevalence of caries remains the highest among oral diseases worldwide and it is the most common chronic disease among children [1]. Untreated dental caries is observed in 28% and 18% of 35–44 and 65+ year-old-people, respectively [2]. Caries’ multifactorial etiology and risk factors have greatly challenged its prevention. The primary factors responsible for dental caries etiology are cariogenic microflora and diet [3]. The microorganisms metabolize fermentable dietary carbohydrates to generate energy for their survival, but at the same time produce organic acids as their waste product. The acids cause demineralization of the tooth that manifest as caries lesions [4]. Furthermore, these microorganisms polymerize the fermentable sugars in our diet to produce extracellular polysaccharides to form oral biofilm (dental plaque) [5]. Among these microorganisms, Gram-positive acid-producing bacteria, specifically Streptococci and Lactobacilli, contribute significantly to dental caries pathogenicity [6,7,8].

The World Health Organization (WHO) recommends limiting sugar intake to less than 10% of total energy consumption in order to decrease the consumption of free sugars [9], which consist of naturally occurring sugars present in food products as well as added sugars. There is growing interest in replacing traditional sugars with polyols due to their low cariogenicity and low calories, in addition to their reduced glycemia and insulinemia [10]. Polyols, otherwise known as sugar alcohols, are not easily broken down by cariogenic microorganisms, and they are less cariogenic substitutes to refined carbohydrates. Isomalt, xylitol, sorbitol, mannitol, lactitol, and maltitol are sugar alcohols commonly used in foods as alternatives to fermentable dietary sugars [11,12]. Previous studies reported that polyols can play a vital role in preventing dental caries. Besides xylitol and sorbitol, which have been widely investigated for their oral health benefit and are presently being used in a variety of products [13,14], Isomalt has been proven to be non-acidogenic and non-cariogenic through several studies [12,15,16,17,18], but its health benefit in oral care products, especially in caries prevention, has not been fully investigated.

Some ingredients that serve as either preservatives or surfactants in oral care products have been found to have certain levels of antimicrobial activities. A cationic quaternary ammonium compound, Cetylpyridinium chloride (CPC), is commonly used in oral hygiene products as a nontoxic and efficient antimicrobial substance that reduces plaque and gingivitis [19,20,21,22,23,24] as well as caries [25]. Another agent, sodium lauryl sulfate (SLS), incorporated into a toothpaste formulation to enhance cleansing through its surface action, has been widely used as surfactant in toothpaste for more than 50 years. It is commonly used at a concentration of 0.5–2% and has been demonstrated to inhibit the growth of a number of microorganisms [26,27,28,29,30].

It is envisaged that the combination of a sugar alcohol and antibacterial agents such as CPC and/or SLS in an oral care formulations might improve the caries prevention effects of fluoride by inhibiting demineralization in this particular formulation. Therefore, in this current study, a multispecies Microbial Caries Model was employed to compare the caries preventive efficacy of toothpaste containing Isomalt, CPC, and SLS (test toothpaste) with that of a standard sodium fluoride toothpaste formulation (1100 ppm fluoride). Our null hypothesis was that toothpaste formulations containing Isomalt would not differ significantly in their inhibition of caries development from formulations without Isomalt.

## 2. Materials and Methods

Healthy human molars that were extracted at different clinics of our school of dentistry (SOD) were collected following the approval (Approval #: HSC20080233N) of the SOD Institutional Review Board. In line with the recommendations from the SOD, the teeth underwent sterilization and were then brushed with pumice slurry using an electric toothbrush (Braun Oral-B Plaque Remover, Proctor and Gamble, Cincinnati, OH, USA), and then examined to verify that no malformations were present. Following that, square enamel samples (3 mm × 3 mm × 2 mm) were produced from each tooth. Subsequently, the enamel and dentin surfaces of the tooth blocks (180) were ground to achieve the planoparallel surfaces necessary for surface microhardness (SMH) measurement. Then, the sides of the blocks were coated with acid-resistant nail varnish.

A Knoop diamond indenter (Tukon 2100; Wilson-Instron, Norwood, MA, USA) was utilized to measure the baseline SMH (SMH_b_) of each tooth block. This was achieved by applying a load of 50 g for 5 s, creating three indentations spaced at least 100 µm apart on the enamel surface. The software then automatically calculated the Knoop hardness numbers and averaged them for each block.

After SMH baseline recording, the 180 blocks were distributed randomly to the treatment groups (*n* = 30) as indicated in Table 1. The toothpaste was applied as slurry (1:3 toothpaste–water ratio mixed to homogeneity). The allocation of tooth blocks to groups was determined by their SMH_b_, resulting in no significant difference in the mean SMH_b_ values among the 6 groups. The tooth blocks in each group were subjected to a high caries-promoting condition under plaque growth in a Microbial Caries Model (MCM) to evaluate the caries control effect of each toothpaste in human tooth enamel.

The experiment was conducted with our MCM, a multiple-chamber continuous flow bacteria culture system [25,31,32,33]. Each group was assigned to an individual chamber, where the samples were secured within an acrylic rod. While in operation, the growth medium (Todd Hewitt Broth) flowed continuously through every chamber and over the samples surfaces to allow biofilm formation. To simulate daily meals, a 10% sucrose solution was provided three times a day for six minutes per session. This method effectively maintained the plaque formation and established a cycle of pH changes involving episodes of demineralization and remineralization. Monitoring of the plaque pH in each chamber was conducted during non-feeding periods. The growth of plaque and development of caries on the sample surfaces were initiated on Day 1 by 12 h circulation of Todd Hewitt broth (THB), previously inoculated with *Streptococcus mutans* and *Lactobacilli casei* culture (broth–inoculum ratio 10:1), through the chambers to promote bacteria adhesion for biofilm formation. The remaining 12 h of the first day were devoted to circulation of bacteria-free THB. Starting on the second day, the sample surfaces covered with biofilm received treatment according to the procedures detailed in Table 2. One group (negative control) received de-ionized distilled water (DDW) treatment, while all other groups were treated with their respective toothpaste, applied both in the morning and evening for 2 min each time. For treatment, the samples were immersed in 150 mL of DDW (negative control) or toothpaste slurry for two minutes, followed by a gentle rinse in sterile PBS. The microbial system was housed in an incubator at 37 °C and the treatments were carried out aseptically within the incubator over a period of 7 days.

On termination of the study after 7 days exposure to plaque growth, post-treatment surface microhardness (SMH_T_) measurement was performed on each tooth block using a similar method as the baseline measurement. The average value (SMH_T_) was calculated from three indentations for each block. For each study group, the percentage change in SMH (%ΔSMH) was calculated relative to SMH_b_ as follows:

%ΔSMH= ([SMH_b_ − SMH_T_] × 100)/SMH_b_

Following the SMH_T_ measurement, a 100 µm thick slice of enamel was sectioned from each sample by cutting perpendicularly to the enamel surface. The slices underwent micro-radiography as described in our previous publication [16]. The resultant microradiographic images were analyzed using TMR2006 version 3.0.0.6 image analysis program (Inspektor Research, Amsterdam, The Netherlands) to quantify the integrated mineral loss (vol%. µm) for each sample as described by de Josselin de Jong [34]. The mean (*n* = 30) mineral loss (∆z) for each experimental group was then calculated.

The statistical analysis was carried out using Stata 11.0 (StataCorp, College Station, TX, USA) statistical software, with significance level pre-determined at *p* < 0.05 for all statistical tests. ANOVA was employed to compare the mean SMH_b_ of all groups. Significant changes in SMH (demineralization) within each study group were identified by comparing the mean (*n* = 30) SMH_b_ and SMH_T_ of the group using paired *t*-test (intra-group comparison). Furthermore, comparison of the six groups (five toothpaste groups and the DDW control) among themselves was conducted with ANOVA and Tukey’s pairwise multiple comparison test based on the mean %ΔSMH and ∆z (vol%.µm) of each group.

## 3. Results

### 3.1. Demineralization Assessment by Surface Microhardness (SMH) Testing

**Figure 1:**
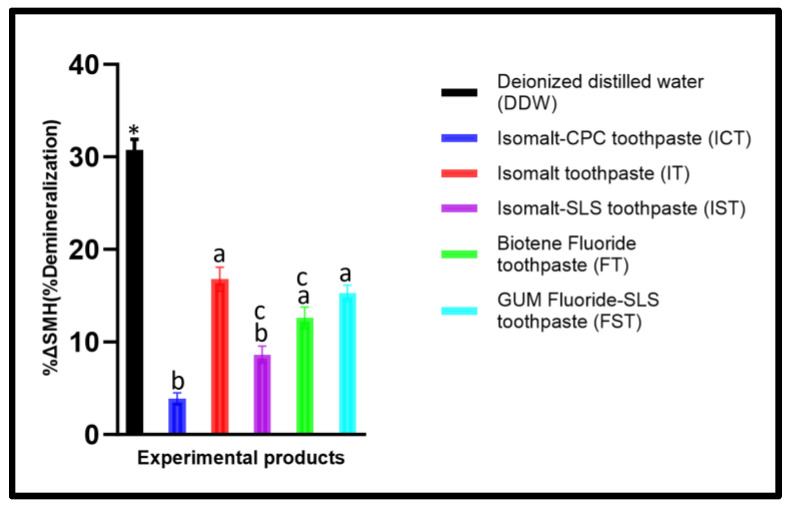
Mean percentage change in surface microhardness (%∆SMH) with each product. * Significantly higher than all other groups; the same letters (a, b, c) mean the products are not significantly different from each other.

Prior to the test, there was no significant difference (*p* > 0.05) in the mean SMH_b_ values across the groups. There was a significant difference (*p* < 0.001) between SMH_b_ and SMH_T_ indicating a significant demineralization across all products, with the exception of Isomalt–CPC toothpaste (ICT). Furthermore, an ANOVA analysis comparing the mean values of %ΔSMH among the groups also showed a significant difference (*p* < 0.001). According to Tukey comparison, the demineralization (%ΔSMH) observed with DDW was significantly greater than with other products (*p* < 0.001) (Figure 1). In comparison to DDW, each toothpaste formulation inhibited demineralization at different percentages (Table 3). Comparing ICT with the other products, there was significantly greater demineralization in Isomalt toothpaste (IT) (*p* < 0.001), Biotene fluoride toothpaste (FT) (*p* < 0.001), and GUM fluoride–SLS toothpaste (FST) (*p* < 0.001) but ICT and IST did not differ (*p* = 0.132) significantly (Figure 1). Also, there was no significant difference between IST and FT (*p* < 0.063) (Figure 1). There was no difference among these three toothpaste formulations (IT, FT, and FST; *p* > 0.05).

### 3.2. Demineralization Assessment Using Transverse Microradiography (TMR)

**Figure 2:**
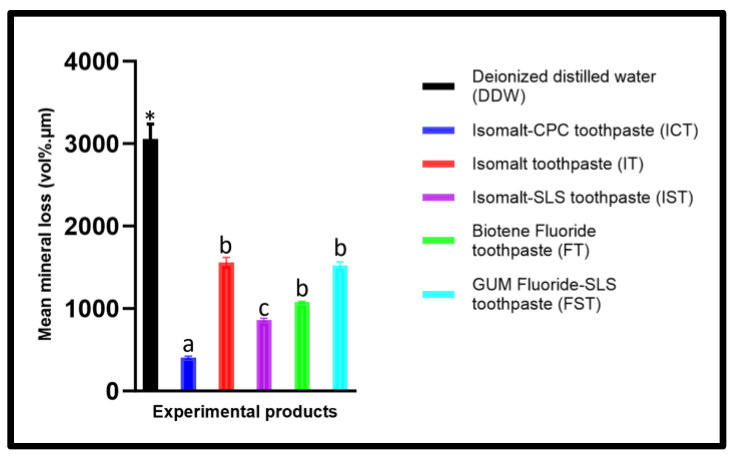
Mean mineral loss observed with each group. * Significantly greater than other groups; ^a,c^ Significantly different from each other; ^b^ no significant difference from each other.

**Figure 3:**
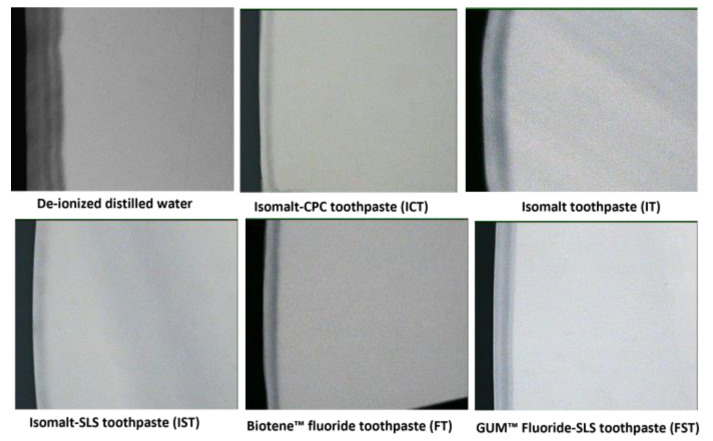
Representative microradiographic images from each group displaying the variation in demineralization across the groups.

When the groups were compared using the mean values of the amount of mineral loss (∆z), there was significant difference (*p* < 0.0001) between the groups (ANOVA). Significantly (Tukey’s; *p* < 0.001) greater demineralization (Δz) was observed with DDW compared to the toothpastes (Figure 2). In comparison to DDW, all toothpastes prevented ∆z but at varying levels (Table 4). All comparisons of the toothpastes with one another were statistically significant (Tukey’s; *p* < 0.001), with the exception of IT vs. FST (*p* = 0.9995) and IT vs. FT (*p* = 0.4209). Microradiographic images in Figure 3 highlights the differences in demineralization levels across the treatment groups. The greatest demineralization was observed in samples from the DDW group, leading to loss of the intact surface layer, typica characteristics of an initial caries lesion, observed in all other groups. Actually, an intact layer was developed in the DDW control sample within four days, but as we proceeded to 7 days to enable lesions to develop in other groups, the lesions in DDW groups became so large and the intact layer disappeared. The least amount of demineralization was observed in ICT and IST, the toothpaste formulations that contain 10% Isomalt, 1100 ppm fluoride and 0.05% CPC (ICT) or SLS (IST).

## 4. Discussion

Although dental caries is a preventable disease, it continues to be a widespread chronic disease worldwide, with a prevalence rate of 35% across all age groups [1]. Fluoride interventions have provided the most reliable benefit in combating caries formation and progression [35], yet caries lesions still develop in individuals at high risk [2,4,14]. Thus, it is essential to explore alternative therapies that can be combined with fluoride for enhanced effectiveness in caries management [36,37]. The present study assessed the efficacy of the toothpaste that combined 10% Isomalt, 1100 ppm fluoride, and 0.05% CPC in preventing caries development and compared it with formulations without Isomalt. The research was carried out utilizing a multispecies MCM acting as an artificial mouth, which facilitated the formation of caries-causing biofilm, while mimicking natural oral environment as much as possible [25,31,32,33]. This model demonstrated a high caries risk condition in which the samples were subjected to the biofilm induced and modulated demineralization–remineralization cycles by the application of the intervention products in the presence of dental biofilm that is regularly nourished with 10% sucrose, without any toothbrushing involved [31]. The outcomes of this study rejected the null hypothesis as the group containing Isomalt and CPC (ICT) consistently revealed higher effectiveness in inhibiting demineralization when compared to the other groups, with similar trends observed with both SMH (%ΔSMH) and TMR (Δz) assessments (Figure 1 and Figure 2). In assessment with SMH, an effectiveness comparable to that of ICT was observed with IST in which the CPC was replaced with SLS, but the protection was quantitatively reduced relative to ICT (Figure 1). The superior performance of the ICT when compared to comparator toothpastes in inhibiting tooth demineralization was additionally proven when statistical comparison of the pretreatment and the post-treatment SMH of the enamel in each product group (intra-group comparison) showed significant demineralization in all groups, but not in ICT. This superior performance in protecting the tooth’s surface against caries development was neither observed with IT that contains only Isomalt and fluoride nor with FST that contains only fluoride and SLS (Figure 1 and Figure 2). Thus, the significantly greater effectiveness observed with ICT and IST can be attributed to the synergistic effects of Isomalt and CPC or SLS. This synergy is feasible considering the known facts that Isomalt has been proven to be non-acidogenic and non-cariogenic through several studies [11,12,13,14,15]. Furthermore, it was also reported that Isomalt could promote caries preventions by binding to calcium [11,12,15]. An in vivo study showed that the difference in relative remineralization between the group using daily 10% Isomalt toothpaste and the control group was significant after the first month [12]. Previous studies reported that different sugar alcohols inhibit both the proliferation of and acid production by S. mutans in the presence of glucose [38,39]. Cetylpyridinium chloride (CPC) has long been used in oral hygiene products to reduce plaque and gingivitis due to its safe and effective antimicrobial actions [19,20,21,22,23,24]. CPC applied as nanoemulsion has been demonstrated as an effective caries-preventive therapy [25,32,33]. Moreover, it has been observed that CPC affects the progression and the maturation of the dental plaque through its ability to reduce both the size and the connectivity in the bacterial network, particularly the bacteria that cause periodontal disease [18]. CPC acts as an inhibitor of fructosyltransferases, an enzyme that aids the bacteria in synthesizing fructans from sucrose and contributes to the development of caries [25]. In a similar manner, sodium lauryl sulfate (SLS), a commonly used surfactant in toothpaste, has been shown to inhibit the development of several microorganisms, including cariogenic bacteria [26,27,28,29,30]. The potential of SLS to adsorb and penetrate through the bacterial cell membrane to interact with components of the bacterial cytoplasm is the basis of its antimicrobial action. This penetration of SLS into the bacterial cell membrane increases the membrane permeability, leading to leakage of intracellular components and, ultimately, cell lysis [28]. SLS could inhibit plaque formation by disrupting the biofilm [27,28,29]. Furthermore, SLS could decrease the surface tension, which also smoothens the surface, challenging the bacterial adherence by glucans [26]. However, there are concerns that SLS could cause irritation of the skin and mucosa [40] and increase the pain in recurrent aphthous stomatitis [41]. Therefore, the concentration of SLS could be reduced to a minimum by adding other active antibacterial agents. It is noteworthy that the possible synergistic relationships among the ingredients in formulations were neither seen between fluoride and Isomalt (IT) nor between fluoride and SLS (FST). Thus, combining Isomalt with either CPC or SLS enhanced its effectiveness in preventing caries than when combined with only fluoride.

It was not surprising that each of the toothpaste formulations investigated showed a significant ability to inhibit caries development, although to a different percentage (Table 3 and Figure 3), given that each tested formulation has 1100 ppm of fluoride. It has been well established with a high level of supporting evidence that fluoride formulations prevent caries development through tooth demineralization inhibition and retardation of the progression of initial caries [35,42,43,44,45]. However, only toothpastes combining Isomalt and CPC (ICT) or Isomalt and SLS (IST) were more effective at inhibiting demineralization than toothpaste with fluoride alone (FT), with ICT and IST achieving 87% and 72% inhibition (% reduction of mineral loss), respectively, as opposed to the 65% inhibition achieved by FT (Table 3). It is strongly believed that there is a likely synergizing relationship between Isomalt and CPC or SLS, as previously mentioned in the preceding paragraph. It is well known that caries prevention by fluoride is dose-dependent, and in a poor oral hygiene condition, as simulated in this study, the standard concentration (1100–1500 ppm) did not yield significant caries prevention [46,47].

Although the Microbial Caries Model used in this study tried to mimic the oral cavity as closely as possible, this present study has certain limitations, one of which is being an in vitro study where many confounding variables that would be encountered in the oral environment were controlled. However, the findings garnered from the present in vitro study can be used as a foundation for developing further studies aimed at testing the toothpaste in clinical trials to confirm that the isomalt-containing toothpastes are more effective in preventing dental caries. Another limitation of the study is the absence of a CPC control group to show the effect of CPC without Isomalt.

## 5. Conclusions

Within the limits of this in vitro study, all the tested toothpaste formulations showed a significant ability to inhibit tooth surface demineralization, but toothpaste formulation combining 10% Isomalt, 1100 ppm fluoride, and 0.05% cetylpyridinium chloride showed the most effectiveness. Thus, toothpaste with 10% Isomalt, 1100 ppm fluoride, and 0.05% CPC demonstrated greater efficacy in inhibiting dental caries development amid dental plaque compared to toothpaste with only 1100 ppm fluoride. This result needs to be validated by randomized controlled clinical trials.

## Figures and Tables

**Table 1:** Experimental groups and the toothpaste formulations.

Toothpaste Composition	De-Ionized Distilled Water (DDW)	Isomalt-CPC Toothpaste (ICT)	Isomalt Toothpaste (IT)	Isomalt-SLS Toothpaste (IST)	Biotene Fluoride Toothpaste (FT)	GUM Fluoride-SLS Toothpaste (FST)
Fluoride		1100 ppm	1100 ppm	1100 ppm	1100 ppm	1100 ppm
Isomalt		10 %	10 %	10 %	-	-
CPC		0.05%	-	-	-	-
SLS		-	-	Yes	-	Yes

**Table 2:** Treatment schedule for Artificial Mouth System for this study.

Day	Time	Treatment
Day 1	8:00	Circulation of bacteria-free Todd Hewitt Broth (THB) starts.
	10:00–11:00	Bacteria-inoculated THB is circulated for 12 h (adhesion phase)
	11:00	Circulation of bacteria-free THB re-starts.
	20:00	Sucrose circulation for 6 min
	20:06 till next morning	Circulation of bacteria-free THB re-starts.
Day 2–Day 7	7:00	2 min treatment with toothpaste.
	7:02	Circulation of bacteria-free THB re-starts.
	8:00	Sucrose circulation for 6 min
	8:06	Circulation of bacteria-free THB re-starts.
	14:00	Sucrose circulation for 6 min
	14:06	Circulation of bacteria-free THB re-starts.
	19:00	2 min treatment with toothpaste.
	19:02	Circulation of bacteria-free Todd Hewitt Broth (THB) re-starts.
	20:00	Sucrose circulation for 6 min
	20:06	Circulation of bacteria-free THB re-starts.

**Table 3:** Mean percentage change in surface microhardness (%∆SMH) and % demineralization reduction by each toothpaste relative to de-ionized distilled water (DDW). CPC—Cetylpyridinium chloride.

Treatment Groups	%∆SMH (% Demineralization)	% Reduction in Demineralization Relative to Control (DDW)
Deionized distilled water (DDW)	30.8 ± 1.1	-
Isomalt-CPC toothpaste (ICT)	3.9 ± 0.6	88
Isomalt toothpaste (IT)	16.8 ± 1.3	45
Isomalt-SLS toothpaste (IST)	8.6 ± 0.9	72
Biotene Fluoride toothpaste (FT)	12.6 ± 1.2	59
GUM Fluoride-SLS toothpaste (FST)	15.3 ± 0.9	50

**Table 4:** Mean (±SD) mineral loss in each group and the % demineralization reduction (as measured by mineral loss) by each toothpaste relative to the control group. CPC—cetylpyridinium chloride.

Treatment Groups	Mean Mineral Loss ± SD	% Reduction in Mineral Loss Relative to Control (DDW)
Deionized distilled water (DDW)	3059.33 ± 178.33	-
Isomalt-CPC toothpaste (ICT)	404.67 ± 18.89	87
Isomalt toothpaste (IT)	1557.33 ± 61.67	49
Isomalt-SLS toothpaste (IST)	863.33 ± 20.53	72
Biotene Fluoride toothpaste (FT)	1077.27 ± 11.60	65
GUM Fluoride-SLS toothpaste (FST)	1520 ± 47.77	50

## Data Availability

The data presented in this study are available upon reasonable request from the corresponding author (B.A.).

## References

[1] Kassebaum N.J., Bernabé E., Dahiya M., Bhandari B., Murray C.J.L., Marcenes W. (2015). Global burden of untreated caries: A systematic review and meta regression. J. Dent. Res..

[2] Centers for Disease Control and Prevention Preventing Dental Caries with Community Programs. http://www.cdc.gov/OralHealth/publications/factsheets/dental_caries.htm.

[3] Bowden G.H. (2000). The microbial ecology of dental caries. Microb. Ecol. Health Dis..

[4] World Health Organization (1994). Fluorides and oral health. Report of the WHO Expert Committee on Oral Health Status and Fluoride Use. World Health Organ. Tech. Rep. Ser..

[5] Banas J.A. (2004). Virulence properties of *Streptococcus mutans*. Front. Biosci..

[6] Kleinberg I. (2002). A mixed-bacteria ecological approach to understanding the role of the oral bacteria in dental caries causation: An alternative to *Streptococcus mutans* and the specific-plaque hypothesis. Crit. Rev. Oral Biol. Med..

[7] Gao X.J., Fan Y., Kent R.L., Van Houte J., Margolis H.C. (2001). Association of caries activity with the composition of dental plaque fluid. J. Dent. Res..

[8] Aranibar Quiroz E.M., Lingstrom P., Birkhed D. (2003). Influence of short-term sucrose exposure on plaque acidogenicity and cariogenic microflora in individuals with different levels of mutans streptococci. Caries Res..

[9] World Health Organization Guideline: Sugars Intake for Adults and Children. https://www.who.int/publications/i/item/9789241549028.

[10] Rice T., Zannini E., Arendt E.K., Coffey A. (2020). A review of polyols—Biotechnological production, food applications, regulation, labeling and health effects. Crit. Rev. Food Sci. Nutr..

[11] Imfeld T. (1993). Efficacy of sweeteners and sugar substitutes in caries prevention. Caries Res..

[12] Featherstone J.D.B. (1994). Effects of Isomalt sweetener on the caries process: A review. J. Clin. Dent..

[13] ALHumaid J., Bamashmous M. (2022). Meta-analysis on the effectiveness of xylitol in caries prevention. J. Int. Soc. Prevent. Communit. Dent..

[14] Bader J.D., Vollmer W.M., Shugars D.A., Gilbert G.H., Amaechi B.T., Brown J.P., Laws R.L., Funkhouser K.A., Makhija S.K., Ritter A.V. (2013). Results from the Xylitol for Adult Caries Trial (X-ACT). J. Am. Dent. Assoc..

[15] Takatsuka T., Exterkate R.A.M., ten Cate J.M. (2008). Effects of Isomalt on enamel de- and remineralization, a combined in vitro pH-cycling model and in situ study. Clin. Oral Investig..

[16] Amaechi B.T., AbdulAzees P.A., Mohseni S., Luong M.N., Lin C.Y., Restrepo-Ceron M.C., Kataoka Y., Omosebi T.O., Kanthaiah K. (2024). Caries preventing efficacy of new Isomalt-containing mouthrinse formulations: A microbial study. BDJ Open..

[17] Imfeld T.N. (1983). Non-nutritive sweeteners, sugar substitutes, and confectionery products. Identification of Low Caries Risk Dietary Components.

[18] Ichikawa T., Yano Y., Fujita Y., Kashiwabara T., Nagao K. (2008). The enhancement effect of three sugar alcohols on the fungicidal effect of benzethonium chloride toward *Candida albican*. J. Dent..

[19] Schaeffer L.M., Szewczyk G., Nesta J., Vandeven M., Du-Thumm L., Williams M.I., Arvanitidou E. (2011). In vitro antibacterial efficacy of cetylpyridinium chloride-containing mouthwashes. J. Clin. Dent..

[20] Latimer J., Munday J.L., Buzza K.M., Forbes S., Sreenivasan P.K., McBain A.J. (2015). Antibacterial and anti-biofilm activity of mouth rinses containing cetylpyridinium chloride and sodium fluoride. BMC Microbiol..

[21] Teng F., He T., Huang S., Bo C., Li Z., Chang J., Liu J., Charbonneau D., Xu J., Li R. (2016). Cetylpyridinium chloride mouth rinses alleviate experimental gingivitis by inhibiting dental plaque maturation. Int. J. Oral Sci..

[22] Haps S., Slot D.E., Berchier C.E. (2008). The effect of cetylpyridinium chloride-containing mouth rinses as adjuncts to toothbrushing on plaque and parameters of gingival inflammation: A systematic review. Int. J. Dent. Hyg..

[23] Gunsolley J.C. (2010). Clinial efficacy of antimicrobial mouthrinses. J. Dent..

[24] Langa G.P.J., Muniz F.W.M.G., Costa R.D.S.A., da Silveira T.M., Rösing C.K. (2021). The effect of cetylpyridinium chloride mouthrinse as adjunct to toothbrushing compared to placebo on interproximal plaque and gingival inflammation-a systematic review with meta-analyses. Clin. Oral Investig..

[25] Lee V.A., Karthikeyan R., Rawls H.R., Amaechi B.T. (2010). Anti-cariogenic effect of a cetylpyridinium chloride-containing nanoemulsion. J. Dent..

[26] Barkvoll P., Rolla G., Svendsen K. (1989). Interaction between chlorhexidine digluconate and sodium lauryl sulfate in vivo. J. Clin. Periodontol..

[27] Moran J., Addy M., Newcombe R. (1988). The antibacterial effect of toothpastes on the salivary flora. J. Clin. Periodontol..

[28] Nordstrom A., Mystikos C., Ramberg P., Birkhed D. (2009). Effect on de novo plaque formation of rinsing with toothpaste slurries and water solutions with a high fluoride concentration (5000 ppm). Eur. J. Oral Sci..

[29] Giertsen E., Scheie A.A., Rolla G. (1989). Plaque inhibition by a combination of zinc citrate and sodium lauryl sulfate. Caries Res..

[30] Sälzer S., Rosema N.A., Martin E.C., Slot D.E., Timmer C.J., Dörfer C.E., van der Weijden G.A. (2016). The effectiveness of dentifrices without and with sodium lauryl sulfate on plaque, gingivitis and gingival abrasion--a randomized clinical trial. Clin. Oral Investig..

[31] Amaechi B.T., Abdul Azees P.A., Farah R., Movaghari Pour F., Dillow A.M., Lin C.Y. (2023). Evaluation of an Artificial Mouth for Dental Caries Development. Microorganisms.

[32] Ramalingam K., Amaechi B.T., Ralph R.H., Lee V.A. (2012). Antimicrobial activity of nanoemulsion on cariogenic planktonic and biofilm organisms. Arch. Oral Biol..

[33] Karthikeyan R., Amaechi B.T., Rawls H.R., Lee V.A. (2011). Antimicrobial activity of nanoemulsion on cariogenic *Streptococcus mutans*. Arch. Oral Biol..

[34] de Josselin de Jong E., ten Bosch J.J., Noordman J. (1987). Optimised microcomputer guided quantitative microradiography on dental mineralised tissue slices. Phys. Med. Biol..

[35] Tenuta L.M.A., Cury J.A. (2010). Fluoride: Its role in dentistry. Braz. Oral Res..

[36] Rethman M.P., Beltrán-Aguilar E.D., Billings R.J., Burne R.A., Clark M., Donly K.J., Hujoel P.P., Katz B.P., Milgrom P., Sohn W. (2011). Nonfluoride caries-preventive agents: Executive summary of evidence-based clinical recommendations. American Dental Association Council on Scientific Affairs Expert Panel on Nonfluoride Caries-Preventive Agents. J. Am. Dent. Assoc..

[37] Twetman S. (2010). Treatment protocols: Nonfluoride management of the caries disease process and available diagnostics. Dent. Clin. N. Am..

[38] Dhar V., Tinanoff N. (2016). Update on Sugar Alcohols and Their Role in Caries Prevention. Decis. Dent..

[39] Milgrom P., Söderling E.M., Nelson S., Chi D.L., Nakai Y. (2012). Clinical evidence for polyol efficacy. Adv. Dent. Res..

[40] Tupker R.A., Vermeulen K., Fidler V., Coenraads P.J. (1997). Irritancy testing of sodium laurate and other anionic detergents using an open exposure model. Skin Res. Technol..

[41] Alli B.Y., Erinoso O.A., Olawuyi A.B. (2019). Effecct of sodium lauryl sulfate on recurrent aphthous stomatitis: A systemic review. J. Oral Pathol. Med..

[42] Marinho V.C., Higgins J.P., Sheiham A., Logan S. (2003). Fluoride toothpastes for preventing dental caries in children and adolescents. Cochrane Database Syst. Rev..

[43] Marinho V.C. (2009). Cochrane reviews of randomized trials of fluoride therapies for preventing dental caries. Eur. Arch. Paediatr. Dent..

[44] Marinho V.C. (2008). Evidence-based effectiveness of topical fluorides. Adv. Dent. Res..

[45] Marinho V.C., Higgins J.P., Sheiham A., Logan S. (2004). One topical fluoride (toothpastes, or mouthrinses, or gels, or varnishes) versus another for preventing dental caries in children and adolescents. Cochrane Database Syst. Rev..

[46] Nordström A., Birkhed D. (2010). Preventive effect of high-fluoride dentifrice (5000 ppm) in caries-active adolescents: A 2-year clinical trial. Caries Res..

[47] Clarkson J.E., Ellwood R.P., Chandler R.E. (1993). A comprehensive summary of fluoride dentifrice caries clinical trials. Am. J. Dent..

